# Sirenomelia in Twin Pregnancy: A Case Report

**DOI:** 10.7759/cureus.48040

**Published:** 2023-10-31

**Authors:** Neha Agrawal, Sonal Prasad, Deepika Manocha, Neeru Malik

**Affiliations:** 1 Obstetrics and Gynaecology, Dr. Baba Saheb Ambedkar Hospital and Medical College, New Delhi, IND

**Keywords:** renal agenesis, anal imperforation, absent external genitalia, mermaid syndrome, caudal regression syndrome, sirenomelia

## Abstract

Sirenomelia is a rare congenital disorder that was once thought to be a severe case of caudal regression but is now thought to be entirely separate. It is often referred to as the "mermaid syndrome" because it causes the lower limbs to atrophy to varying degrees, giving the impression of a mermaid's tail or fin. The syndrome is often viewed as fatal due to the accompanying visceral deformities.

Our case was a live born, delivered at term by caesarean section, to a 30-year-old third gravida having twin pregnancy. Examination of the baby revealed caudal dysgenesis with fusion of lower limbs, non-identifiable external genitalia and anus. The infant survived for 11 hours after birth. We report this case due to their rarity and term live birth.

While sirenomelia is uncommon, the absence of distinct lower limbs on ultrasonography in the presence of oligo or anhydramnios may prompt consideration of the diagnosis of sirenomelia.

## Introduction

The mermaid syndrome, also known as sirenomelia, is a rare, fatal multi-system congenital syndrome. The most common findings are lower limb fusion into a single extremity, deformities of the sacrum and pelvic girdle, absence of external genitalia, absence of anal patency, and lethal renal abnormalities [1]. These infants frequently display Potter's facies and pulmonary hypoplasia as a result of the oligohydramnios that result from renal agenesis [1]. In the literature, 300 cases have been described and 15% of these cases are twins most frequently monozygotic [1]. The incidence of sirenomelia is 0.99 per 100,000 live births, with stillbirths accounting for around one-third of cases [2]. Most of the time, the sex is unknown. In babies with sirenomelia, sex is determined by chromosomal or gonadal sex [2]. With a male-to-female ratio of 2.7:1, there is a male predominance [1]. Additionally, monozygotic twin births appear to have a 100-fold increase in the prevalence of mermaid syndrome [1]. The syndrome is non-compatible with life due to non-functioning kidneys [1]. Sirenomelia can be differentiated from caudal regression syndrome due to the presence of a single umbilical artery which is a remnant of the vitelline artery complex arising from the aorta [3,4]. However, in caudal regression syndrome, there are two umbilical arteries, the presence of functioning kidneys and two hypoplastic lower limbs [5]. Sirenomelia is also strongly associated with maternal pregestational diabetes [6].

## Case presentation

**Figure 1:**
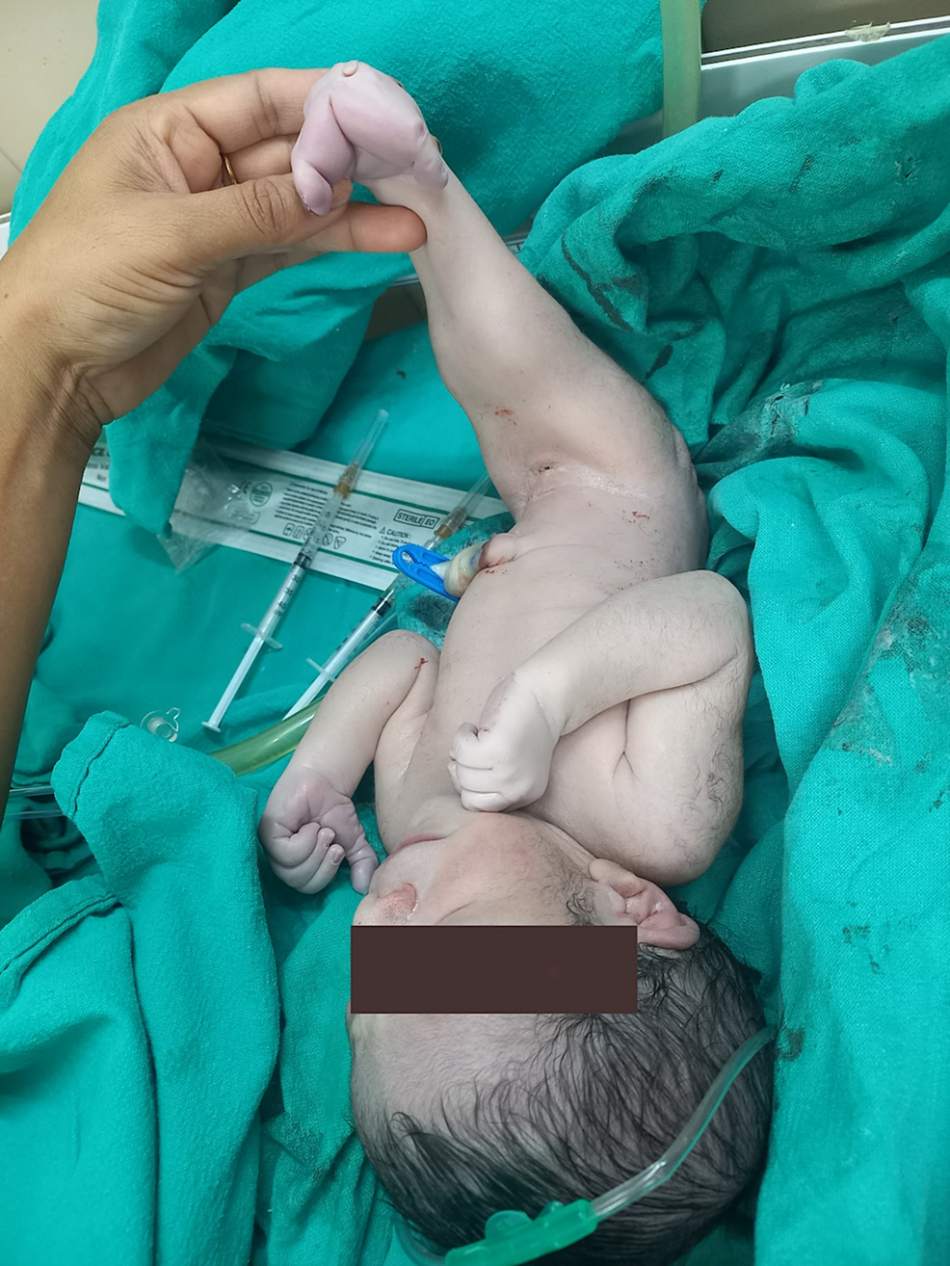
Photograph of the baby showing fusion of lower limbs and absent external genitalia

A 30-year-old female with G3P2L2 was booked for antenatal checkup in a dispensary. She had previous two full-term normal vaginal delivery of both male child. Her first ultrasound was done at 24 weeks which showed twin monochorionic pregnancy. Fetus A was normal, while fetus B had absent kidneys and bladder. The weight of fetus B was 30% less as compared to fetus A. The female was counselled. She wanted to continue her pregnancy as one fetus was normal. An oral glucose tolerance test was done which was normal. The female was referred to our medical college for further management. Ultrasound done in our hospital at 37 weeks showed Fetus A of 2.8 kg in breech presentation with all parameters corresponding to 37 weeks, while Fetus B of 1.7 Kg, with all parameters corresponding to 31 weeks and nil liquor. No comment was made on the spine due to poor visualisation of the fetus due to anhydramnios. This was a case of discordant twin. The patient was taken for caesarean section because of term pregnancy with the first twin in breech presentation. Twin A was a live male baby of 2.5 kg. Twin B was a sirenomelic baby of 1.6 kg with an unknown gender which cried on stimulation (Figure 1).

**Figure 2:**
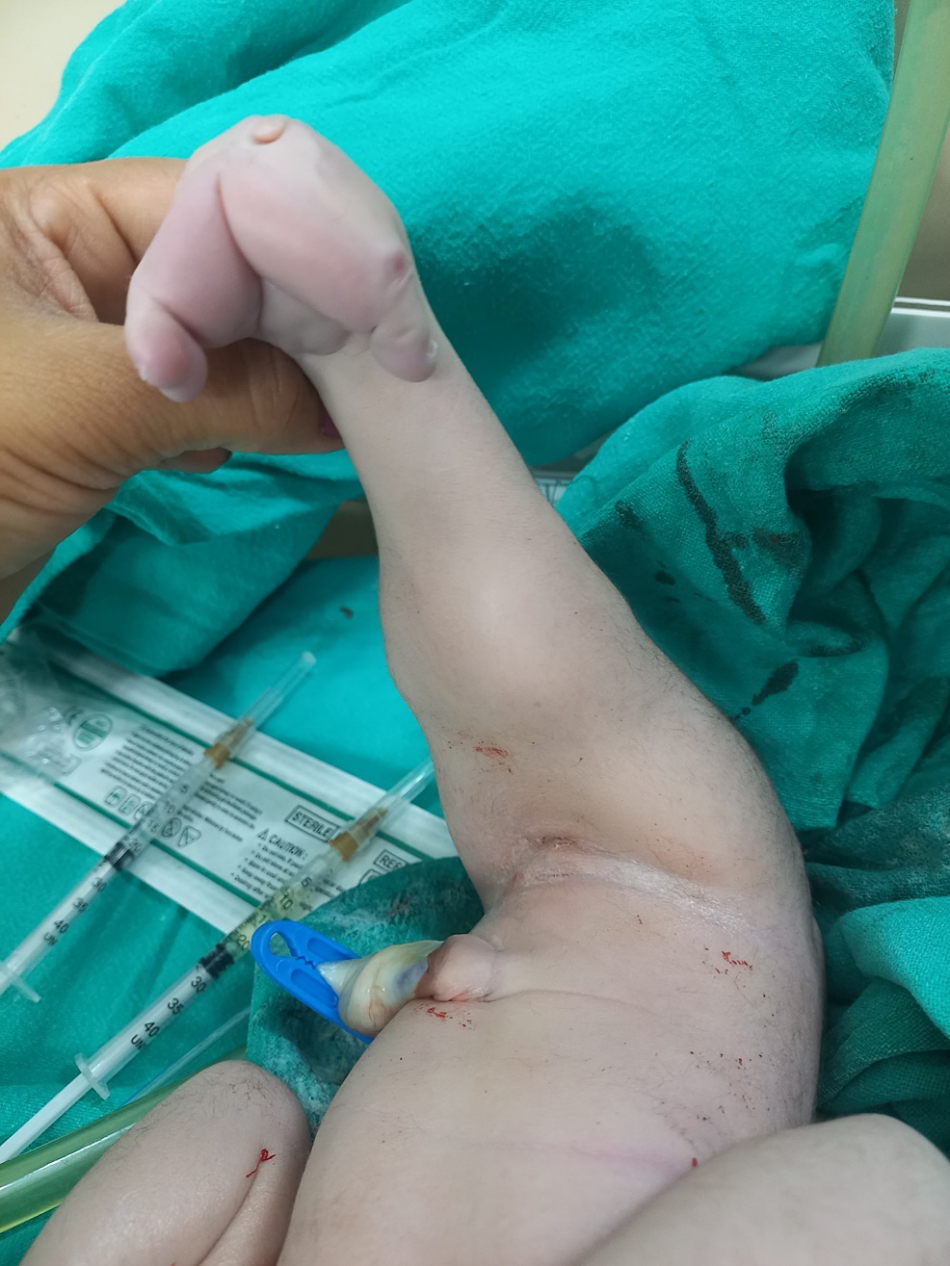
Photograph of the baby showing absence of external genitalia and malformed toes

**Figure 3:**
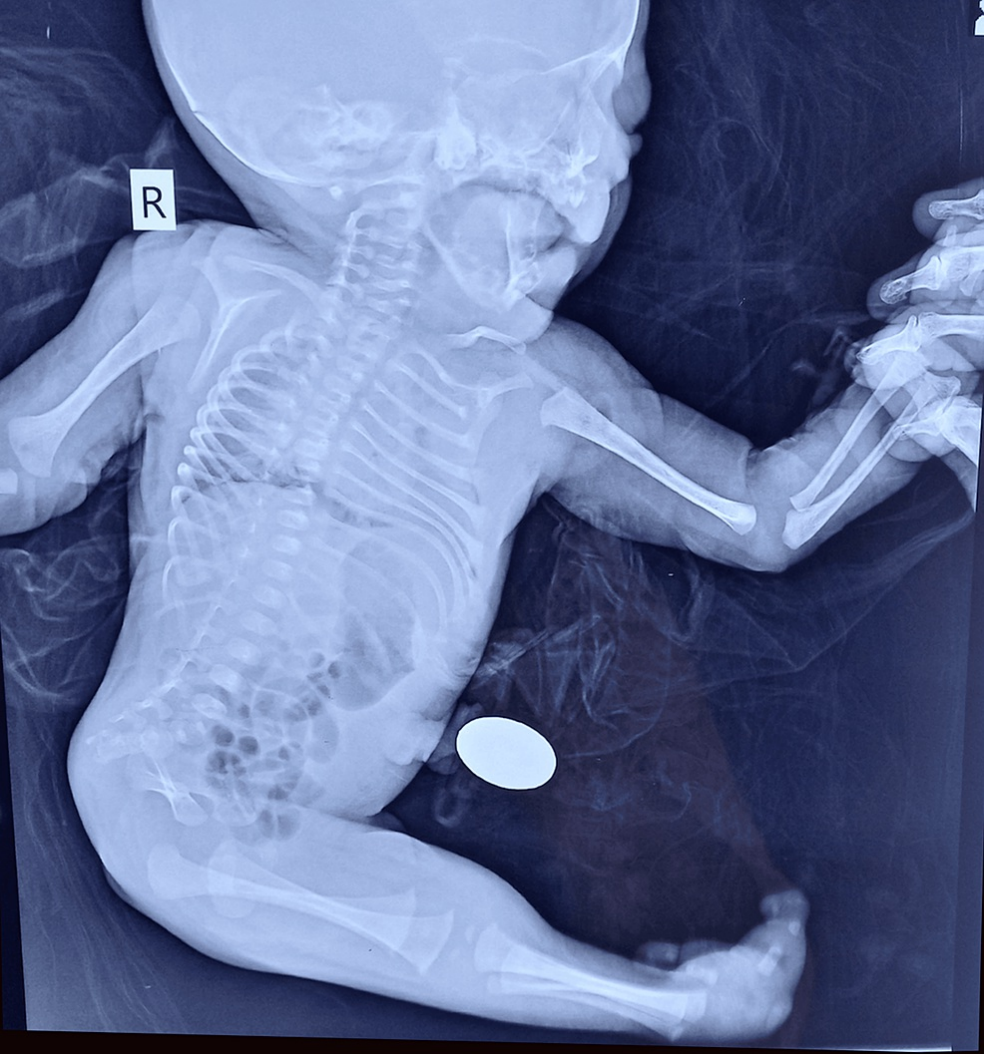
X-ray of sirenomelia baby

Physical examination of Twin B at birth showed the infant had fused lower limbs with malrotated toes, no external genitalia (Figure 2), no anal patency and the face showed a receding chin. X-ray showed two femurs, two tibias, only one fibula, sacral agenesis, and malformation of the pelvis (Figure 3). Ultrasound of the baby was not done. Soon after birth, the baby developed respiratory distress, requiring oxygen via m-CPAP. The baby survived for 11 hours and died due to respiratory distress with refractory respiratory shock.

## Discussion

The origin of the mermaid legend by the Greeks and Romans probably lies in ancient observations of certain types of human anomalies. The term mermaid evokes more of mythology than of pathology. Sirenomelia is also known as the mermaid syndrome [1]. The existence of such human sireniform babies was first documented in the first century AD [7]. Ballantyne (I904) classed sirenomelia as monopodia or sympodia, depending on whether the lower limbs were fused completely or only partially. He acknowledged that these infants were stillborn or died just after birth as these babies do not have kidneys. He observed the significant internal pelvic anomalies linked to the limb deformity and appropriately defined the potential limb deformities [8]. Duhamel made the hypothesis that siren morphology and anal abnormalities may perhaps be the two extremes of one complete syndrome of an embryonal anomaly in the creation of the caudal area in 1961. "Syndrome of caudal regression" is how he described it [7].

Classification has been challenging because of the large range of limb phenotypes. However, due to the presence of skeletal components in the thigh and leg, Stocker and Heifetz categorised sirenomelia from type I to type VII (Stocker and Heifetz, 1987) [9]. The limb's bones - two femurs, two tibias, and two fibulae - are present in type I, the mildest version. Soft tissues alone are united. Two femurs and two tibias are present in type II, but the fibulae are fused, and there are no fibulae at all in type III. Types IV and V both have partially fused femurs, however, type IV differs from type V in that it has fused fibulae whereas type V does not. A fused femur and fused tibia are seen in type VI. The only bone seen in type VII, the most severe variant, is a fused femur; there are no signs of legs or feet [9].

Sirenomelia can be first diagnosed at 13 weeks by a transvaginal ultrasound [10]. Anhydramnios may restrict the diagnosis in the late second and third trimesters because renal agenesis prevents urine production. The fact that the femurs move uniformly during the ultrasound could be a sign. Ultrasound can also demonstrate absent kidneys. A single umbilical artery and a missing bladder are possible additional findings [10].

Though it has been called "fused lower limbs," this is actually more likely to be a result of the lower limb buds failing to undergo blastogenesis [4]. There are two different aetiology theories. The "vascular steal theory" by Stevenson et al. indicates that the blood is returning to the placenta through a single large vessel derived from the vitelline artery below the diaphragm rather than through paired umbilical arteries from the iliac arteries [4]. This diverts blood flow from the developing caudal structures (vascular steal) thus tissues distal to this site are malformed [4]. The alternative theory is the primary development defect of blastogenesis at the caudal eminence. The caudal eminence contributes to the production of mesenchyme for the lower limb buds, perineum, somites, and vertebrae. Caudal dysgenesis and sirenomelia in extreme forms can result from interfering with morphogenetic processes at the caudal eminence [11]. According to additional theories by Davies et al., sirenomelia is caused by damage to the caudal mesoderm between 28 and 32 days of foetal development [12].

A case study by Di Lorenzo et al. shows sirenomelia in monozygotic twins where one twin was a normal male and the other was sirenomelia. The sirenomelic baby in the study was born at term, weighing 2.7 kg having normal facies, two femurs, two tibias and two fibula. The sirenomelic infant was sedated and allowed to die [1]. Another study by Davies et al. shows a monozygotic twin with the first twin being female and a second sirenomelic baby of 1.5 kg who had normal facies with a single lower extremity terminating in a single big toe. The femur was fused and the lower leg had a single bone, a fused tibia. The infant survived for five days and died following a seizure [12]. Another study by Das et al. shows a full-term sirenomelic baby of 1.9 kg who had flattened facies, two femurs, and two tibias but absent sacrum and absent fibula. The infant expired one hour after delivery [3]. Our study is unique as this is a case of sirenomelia in a monozygotic twin having discordant growth. In our study, the twins are delivered at term where one infant is a healthy male of 2.5 kg who is phenotypically normal. The second twin was a sirenomelic baby of 1.6 kg. The sirenomelic infant had fused lower limbs consisting of two femurs, two tibias, and only one fibula, absent external genitalia, and the face showed a receding chin. The sirenomelic baby survived for 11 hours and died due to respiratory distress. The presence of paired femurs in our patient indicates that the condition is probably caused by failure in lateralization of the lower limbs, arising at approximately four weeks.

Animal models (mice) with sirenomelia have been found to contain mutations in the CYP superfamily [13]. Specifically, CYP26A1 is the receptor for retinoic acid. Due to exposure to high doses of retinoic acid, there could be lower vertebral column abnormalities [14]. The relationship between bone morphogenic protein 7 (BMP7) and twisted gastrulation (Tsg) is another discovery; in mice models, sirenomelia is connected to the loss of BMP7 as well as a full loss or half-dose of Tsg [15]. These studies have not been replicated in humans. In animal models, sirenomelia has been linked to exposure to retinoic acid, cadmium, lithium, diethylpropion, lead, hyperthermia, and radiation [3]. There are also case reports of sirenomelia following cocaine use [16]. In our study, there was no history of any teratogen exposure, and the mother was not diabetic. Prior to the last two decades, sirenomelia was mostly deadly, however, there are a few case reports of patients surviving to their first few years of life. There was renal tissue and enough amniotic fluid to support pulmonary development, according to case reports of survival. To separate the lower extremities, a multidisciplinary surgical approach is performed, including soft tissue expanders, skin flaps, and urine drainage procedures. However little has been reported about long-term outcomes [17-19].

## Conclusions

Sirenomelia is a lethal congenital anomaly. Our case is a unique case of sirenomelia as there is a striking contrast between the affected twin and the spared one. This observation underscores the complexity of ethical considerations in cases involving twins with lethal diseases. In our case, the woman knew about the condition but chose to continue the pregnancy as the other twin was normal. This case serves as a poignant reminder of the ethical complexities inherent in managing rare congenital disorders in twin pregnancies, promoting further reflection on best practices in such challenging clinical scenarios.
